# Patients with Parkinson’s Disease and Myasthenia Gravis—A Report of Three New Cases and Review of the Literature

**DOI:** 10.3390/medicina56010005

**Published:** 2019-12-23

**Authors:** Irina Odajiu, Eugenia Irene Davidescu, Cristina Mitu, Bogdan Ovidiu Popescu

**Affiliations:** 1 Neurology Department, Colentina Clinical Hospital, 020125 Bucharest, Romania; irina.odajiu@yahoo.com (I.O.); cristinaelenam@yahoo.com (C.M.); bogdan.popescu@umfcd.ro (B.O.P.); 2 Department of Clinical Neurosciences, ‘Carol Davila’ University of Medicine and Pharmacy, 020021 Bucharest, Romania; 3 Laboratory of Molecular Biology, ‘Victor Babeș’ National Institute of Pathology, 050096 Bucharest, Romania

**Keywords:** Parkinson disease, Myasthenia gravis, co-occurrence, case report

## Abstract

Neurodegenerative diseases such as Parkinson’s disease (PD)have increasing incidence, due to lifespan expansion. The association between PD and Myasthenia Gravis (MG) is uncommon, and so far, since 1987, 26 cases have been reported. We report here a series of three new cases, two men and one woman with this peculiar combination of conditions, identified in the Neurology Department of Colentina Clinical Hospital. In this article, the pathogenesis of MG in patients with PD is discussed, along with a literature review regarding the co-occurrence of these two neurological diseases.

## 1. Introduction

Among the neurological entities, Parkinson’s disease (PD) has an incidence of approximately eight to 18.6 per 100,000 persons in a year [1], whereas Myasthenia gravis (MG) is encountered even more rarely. Its incidence is around seven to 23 new cases each year, per million persons [2,3,4,5]. Thus, the concomitant diagnosis of PD and MG is exceptionally limited—the first ever published association was in 1987 [6]. We report three new cases of patients diagnosed with PD, who subsequently developed MG.

## 2. Materials and Methods

We performed a systematic review of the literature based on the PubMed database, using the combination of “Parkinson’s disease” and “Myasthenia gravis” as search elements, and identified 26 previous published cases. Further on, we report a series of three new cases with this co-occurrence, that were diagnosed in the Neurology Department of Colentina Clinical Hospital, Bucharest, Romania.

### 2.1. Case 1

A 60 year old male patient, who, in 2008, developed right limb bradykinesia and was subsequently diagnosed with Parkinson’s disease. Currently, he presents motor fluctuations with short OFF motor periods and important dyskinesia during the ON period for most of the day (Hoehn & Yahr st. III), and is undergoing treatment with Levodopa/Carbidopa/Entacapone 150/37.5/200 mg 6 × a day, Ropinirole 16 mg q.d., Rasagiline 1 mg q.d. and Madopar HBS (Levodopa/Benserazide) 100/25 mg q.d. N.B. He is also diagnosedwith Crohn’s disease and underwent treatment with Infliximab, but subsequently developed anti-Infliximab antibodies.

He presented in our clinic in July 2019, complaining of right palpebral ptosis and vertical diplopia, symptoms that had appeared one month before, which improved in the morning and worsened during the day. Neurologic examination revealed generalized dyskinesias, right palpebral ptosis, vertical down-gaze limitation of the left eye, hypomimic facies, mild bilateral bradykinesia and rigidity (left > right). The serum anti – acetylcholine receptor antibody (anti-AchR Ab) level was significantly increased (9.2 nmol/L vs. <0.25–normal range), therefore the patient underwent the test with Neostigmine with dramatic improvement in the palpebral ptosis and oculomotor limitation, highly suggestive for the diagnosis of Myasthenia gravis (Osserman group 1), even though no decrement could be detected on repetitive stimulation. Consequently, the patient started treatment with Neostigmine 60 mg bis in die (b.i.d.), which was well tolerated, improving symptoms.

### 2.2. Case 2

A 69 year old female patient, known to have hypertension and Parkinson’s disease (Hoehn&Yahr stage II) since 2014, when disease started with right upper-limb kinetic disability, is currently treated with Levodopa/Carbidopa/Entacapone 100/25/200 mg 1 tb ter in die (t.i.d.), Pramipexole 1.05 + 0.26 mg and Rasagiline 1 mg 1 tb quaque die (q.d.), without motor fluctuations, but with associated Rapid Eye Movement (REM) sleep disorder.

The patient presented to our clinic in June 2018, complaining of a symptomatology that had appeared over the previous three months, being mostly bothersome during the second part of the day, comprising palpebral ptosis, dysarthria and swallowing problems for solids. Additionally, the patient reported a painful abdominal syndrome of medium intensity, that increased after food intake and during orthostatic position, thus forcingher to adopt a camptocormic posture, which developed in the previous three months. On neurologic examination, the patient presented palpebral ptosis, diminished velopalatine reflexes, dysphagia for solids, lack of tongue movement from side to side with diminished tongue protrusion, bradykinesia (right > left), hypomimic face, limb and axial rigidity, dysarthria, a Quantitative Myasthenia Gravis Test of eight points, Mini Mental State Examination (MMSE)—30/30pts, Montreal Cognitive Assessment (MoCA)—26/30pts. Although there was no decrement on repetitive stimulation (5 Hz) of the abductor digiti minimi (ADM) muscle of the hand and orbicularis oculi muscles, and the serum level of anti-AchR Ab was within normal range, the Neostigmine test was positive.

Therefore, a diagnosis of a generalized form of Myasthenia gravis (Osserman class group 2B) was established and the patient started treatment with Neostigmine 60 mg t.i.d. Apart from that, the dose of Levodopa/Carbidopa/Entacapone was increased to 500 mg/d (5× a day) and the dose of the dopamine agonist was decreased, which led to an improvement in the camptocormic posture and painful abdominal syndrome (interpreted as an OFF period). Melatonin was started for the REM sleep disorder, with the remission of symptomatology.

### 2.3. Case 3

A 64 year old male patient wasdiagnosed with Parkinson’s disease (Hoehn&Yahr st.III) in 2007, controlled by Levodopa/Carbidopa/Entacapone 150/37.5/200 mg t.i.d., Rasagiline 1 mg q.d. and Trihexyphenidyl 2 mg q.d. Heis also diagnosedwith diabetes mellitus and arterial hypertension, and is treatment non-compliant.

The patient presented to our clinic in March 2016, complaining of incomplete palpebral ptosis, which was observed for a period of about six years and aggravated in the previous two years, accompanied by horizontal diplopia, worse in the bilateral lateral gaze. The symptomatology had nonspecific fluctuations(not connected with effort/rest periods). On neurological exam, the patient presented trunk anteroflection, minimal postural and rest tremor of the superior limbs (left > right), hyposmia, limited abduction of both eyes, incomplete bilateral palpebral ptosis (right > left), minimally mydriatic pupils, with a decreased photomotor reflex, hypophonia, global bradykinesia, axial rigidity, and decreased osteotendinous reflexes. The patient underwent repetitive stimulation (3 Hz), which did not show decrement, and the level of serum anti-AchR and anti-muscle specific tyrosine kinase (Anti-Musk) Ab was within normal range. Regardless, we performed the Neostigmine test, with moderate improvement in the palpebral ptosis and diplopia, and a diagnosis of Myasthenia gravis (Osserman group 1) was given. Accordingly, treatment with Trihexyphenidyl (THP) was gradually discontinued and a dopamine agonist was initiated (Ropinirole2 mg/d increased gradually to 8 mg/d) and he also started treatment with Neostigmine 60 mg 1 tb t.i.d, which was later increased to 1 tb × 4/d, with a good improvement in symptomatology.

## 3. Discussion

Since 1987, when the first case report of a patient with PD who later developed MG was reported, 25more cases have been published, of which MG preceded a diagnosis of PD in only two cases. Out of all 26 published cases, the majority were male—19 cases—and only seven were female, with a ratio of approximatively 3:1, ranging between 55 and 95 yearsold, with a medium of 72 years [6,7,8,9,10,11,12,13,14,15,16,17,18,19,20,21,22] (Table 1 and Table 2—review of the literature synopsis).

The time lapse between the two diagnoses, the first being PD, varied between eight months and 20 years. Therefore, the majority of articles mainly concentrated on reporting the course of establishing diagnosis of MG. Mostly, this was based on clinical features, the presence of decrement (1–5 Hz) or increased jitter on electrophysiological studies, the presence of a positive level of anti-AchR antibodies, and a positive edrophonium or neostigmine test.

Regarding the symptomatology, in six cases only head dropwas reported, whereas, in the majority of others, ocular symptoms prevailed along with asthenia—occurring in five cases—dysphagia alone—occurring in one case—associated dysphagia—occurring in nine cases—and dysarthria occurring in another one. Regarding electrophysiological studies, in seven cases decrement was observed, in another three only increased jitter was observed, and no modifications were observed in four cases. In one case there was a myopathic pattern and there are no available data for the other cases. Regarding the use of a cholinesterase inhibitor test, there were seven cases with a positive response to edrophonium, five cases a positive response to neostigmine, in five cases no such test was applied, and in six cases there are no available data. Laboratory determination of serum anti-AchR Ab was performed in all cases, with a positive level in 14 cases and six negative cases, and no data for seven cases. As well as detecting the serum level of anti-AchR Ab, anti-Musk Ab was checked in one case and also proven to be negative, and, in another test, the levels of striational Ab and AchR modulating Ab were positive.

Generally, it is difficult to consider diagnosis of MG in a patient with PD, due to the scarcity of the incidence of these associations. It is also difficult because there are shared symptoms, such as fatigue, muscle weakness, dysarthria, dysphagia and sometimes even diplopia. In the first case described with this association, explanation relied mostly on the adverse effect of Trihexyphenidyl on the neuromuscular junction [6]. Such an adverse effect could have been induced in the case of our third patient as well. In the case reported in 1993, it seemed that pyridostigmine worsened parkinsonian symptoms and its withdrawal led to their improvement and a reduction in the Madopar dosage, which controlled symptomatology. It was considered that an age-related alteration of the blood-brain barrier might occur, leading to the aggravation of PD through the focal increase in acetylcholine activity on the muscarinic receptors and the impairment of the dopamine deficiency state [8]. In recent years, evidence regarding the alteration of the blood–brain barrier came from neuroimaging studies, which exhibited signs of damage in the vicinity of the midbrain, along with diverse deep and cortical white and gray matter regions [23].

Another explanation for the development of MG in a patient with PD can be given by recent evidence of the involvement of immune processes in PD’s pathogenesis. Even though it has been long considered a neurodegenerative disease, several studies have revealed that neuroinflammation is shown on Psitron Emission Tomography (PET) studies in various regions, especially in the basal ganglia and striatum [24], and has autoimmune implications for PD’s pathogenesis. For example, plasma antibodies isolated from PD patients, such as α-synuclein, melanin and monosialotetrahexosyl (GM1) ganglioside, have been proven to recognize dopaminergic cells [25]. As a result, α-synuclein serves as a trigger of this immune response in PD patients, as it seems to be chemo-attractant to neutrophils and monocytes [26]. There are studies demonstrating that α-synuclein pathology may start in the periphery, the enteric nervous system, which is the first component to be affected in PD’s pathological progression, many years before diagnosis [27]. Studies performed on mice established that α-synuclein over-expression serves as a trigger for microglial activation, blood–brain barrier alteration and the recruitment of T and B cells prior to neurodegeneration [28]. Consequently, it is more suggestive that immune cell recruitment and local inflammation serve as components in the process of neurodegeneration and there are no consequences from neuronal death [29].

There is evidence that there are autoreactive T cell lymphocytes, autoantigen presentation and microglial activation in PD patients [30]. These patients tend to have fewer naive T CD^4+^ helper cells and more T helper 2 regulatory cells than healthy controls, which seem to be placed in the neighborhood of blood vessels and neuromelanin-containing dopaminergic neurons [23]. Thus, a dysregulation in the proportion of T helper cells and T regulatory cells contributes to the fact that effector T cells of PD patients produce a greater amount of proinflammatory cytokines [31]. Since there is a dysfunction in T regulatory cells, a proinflammatory environment may prevail in the periphery and in the central nervous system [29]. In sum, a high amount of infiltrating T cells, as well as the sensitivity of dopaminergic neurons to inflammation, may prove that PD’s pathogenesis has features of an autoimmune disorder [29], as MG has.

## 4. Conclusions

Although PD and MG co-occurrence is uncommon, with only case reports or short series of case reports in the literature, it is crucial to not miss diagnosis of MG in a patient with PD, since the treatment implications are essential and crucially influence the prognosis. More basic research needs to be performed to understand the pathogenesis of both diseases, in order to provide more therapeutic options and perhaps change the approach of such patients, whose quality of life is determined by these two neurological diseases, which have an increased disabling impact.

Additional informed consent was obtained from all individual participants for whom identifying information is included in this article.

## Figures and Tables

**Table 1:** Clinical features synopsis (review of literature).

Year	Article	No. of Cases	Sex	Age	PD Duration	PD Clinical Signs	MG Time of Evolution	MG Clinical Signs
1987	Neurology 1987; 37 (5): 832–833; [6]	1	M	55	Five years	Tremor, hypomimia	Five years after PD	Diplopia, bilateral ptosis, muscle weakness of the neck and shoulder muscles
1991	Srp. Arh. Celok. Lek. 1991 Mar-Apr; 119 (3–4): 103–6; [7]	1	M	77	Three years	Tremor, axial rigidity		Bilateral ptosis, diplopia, dysphagia, dysarthria
1993	Clin. Neurol. Neurosurg. 1993; 95 (2): 137–l39; [8]	1	F	62	Eight years	Tremor, hypomimia bradykinesia, Limb rigidity	One year duration, Seven years after PD	Fluctuating left eye ptosis, diplopia, dysphagia, generalized muscle weakness
2003	J. Neurol. 2003; 250: 766–767; [9]	4	3 M; 1 F	76; 62; 68; 61				
2008	Parkinsonism Relat. Disord. 14 (2): 164–165; [10]	1	F	58	Five years	Right superior limb tremor, bradykinesia, rigidity	Six months	Head drop
2009	Movement Disorders, Vol. 24, No. 13, 2009, 2025–2026; [11]	1	M	84	Four years	Right superior limb tremor, bradykinesia, rigidity	Four years after PD	Head drop
2011	Neurologist 2011; 17 (3): 144–146; [12]	1	M	75	Eight years		Eight years after PD	Head drop
2014	The Neurohospitalist. 2014, Vol. 4 (3): 117–118; [13]	2	1 F; 1 M	70; 72	1 two years; 2. one year after MG diagnosis	Right superior limb tremor, bradykinesia, rigidity		1.—generalized form; 2.—ocular form
2014	Neurol. Sci. 2014. 35 (5): 797–799; [14]	1	M	69	Five years	Left superior limb rigidity		Neck flexion weakness
2016	Parkinsonism Relat. Disord. 2016; 28: 166–168; [15]	3	1 F; 2 M	67; 64; 72	1. ten years; 2. four years; 3. two years			1.—asthenia, bilateral ptosis 2.—bilateral ptosis, head drop; 3.—asthenia, bilateral ptosis, diplopia
2016	J. Clin. Anesth. 2016; 34: 350–351; [16]	1	M	68	Two months	Bradykinesia, Dysarthria		
2016	Geriatr. Gerontol. Int. 2016; 16 (4): 528–530; [17]	1	F	90	Nine years	Hypomimia, bradykinesia, rigidity, dysarthria, rest tremor	Nine years after PD	Profound dysphagia
2016	J. Neurol. Disord. 2016, 4:4; [18]	1	M	73	20 years	Hypertonic-akinetic syndrome		Diplopia, bilateral ptosis, dysphagia, muscle weakness of neck, trunk and limbs
2016	J. Am. Geriatr. Soc. 2016; 64 (10): e120–e122; [19]	1	F	76	Five years	Akinesia and slight rigidity		Head drop
2017	J. Neurol. Sci. 2017; 376: 216–218; [20]	1	M	75	Eight months	Hypomimia, Hypophonia, neck rigidity, shuffling gait, right limb bradykinesia		Diplopia, head drop
2018	Nervenartz. 2018: 89 (4): 443–445; [21]	4	M	82; 95; 83; 81	1.—six years; 2.—five years; 3.—five years 4.—seven years			Progressive dysphagia in all four cases
2019	Clin. Neurol. Neurosurg. 2019. 179 1–3; [22]	1	M	73	Eight years	Bradykinesia, rigidity, left limbs rest tremor	Eight years after PD	Asthenia, dysphagia, diplopia

**Table 2:** Review of literature and treatment.

Year	Article	Electromyography (EMG)	Abs	Other MG Tests	PD Treatment	MG Treatment
1987	Neurology 1987; 37 (5): 832–833; [6]	Orbicularis oculi 1–5 Hz decrement	Anti-AchR positive	Positive Edrophonium test	THP	Pyridostigmine 180 mg q.d., THP—stopped, thymectomy
1991	Srp. Arh. Celok. Lek. 1991 Mar–Apr; 119 (3–4): 103–6; [7]	Deltoid and bilateral facial muscle decrement			No data	
1993	Clin. Neurol. Neurosurg. 1993; 95 (2): 137–l39; [8]	No data	Anti-AchR positive	Positive Edrophonium test	THP + Sinemet	1. Pyridostigimine 240–360 mg q.d—led to improvement of ptosis and dysphagia only; 2. Prednisolone 60 mg q.d. until improvement
2003	J. Neurol. 2003; 250: 766–767; [9]	No data			No data	
2008	Parkinsonism. Relat. Disord. 14 (2): 164–165; [10]	Increased jitter in orbicularis oculi, neck extensors	Negative anti-AchR and anti-Musk	Positive Neostigimine test	No PD treatment	1. Pyridostigmine 60 mg q.d.+ Prednisolone 50 mg/d + Azathioprine (AZA) 125 mg q.d.—slight improvement 2. Plasmaferesis six sessions 3. Prednisolone 5 mg + Pyridostimine
2009	Movement Disorders, Vol. 24, No. 13, 2009; 2025–2026; [11]	Increased jitter in left frontalis muscle	Negative anti-AchR	Positive Neostigimine test	No PD treatment	Pyridostigmine 60 mg × 4/d —significant improvement
2011	Neurologist 2011; 17(3): 144–146; [12]	Decrement > 25%	Anti-AchR positive	Not performed	Levodopa + Benserazide (250 mg q.d.)	1. intravenous Immunoglobulin (ivIg)—five days 2. Piridostigmine—180 mg q.d.—led to improvement after three months
2014	The Neurohospitalist 2014, Vol. 4 (3): 117–118; [13]	No data	1.anti-AchR, anti-striational and AchR modulating positive; 2.negative	Not performed	1.—Levodopa + Carbidopa 2.—no treatment	1. Pyridostigmine + AZA—stopped because of adverse reactions then—ivIg 2 g/kg every six weeks for eight years; 2. Pyridostigmine in small dose—led to improvement
2014	Neurol. Sci. 2014. 35 (5): 797–799; [14]	Decrement	Anti-AchR positive	Not performed	No data	Pyridostigmine 60 mg × 4/d + Prednisolone 10 mg—followed by a dose increase of Prednisolone to 75 mg—led to improvement of rheumatoid arthritis
2016	Parkinsonism. Relat. Disord. 2016; 28: 166–168; [15]	Increased jitter in orbicularis, extensor and digitorumcomunis	1. and 2. negative anti-AchR; 3. anti—AchR positive	Positive Neostigimine test	No data	Pyridostigmine 15–30 mg q.d.—in all cases, led to improvement without further progression
2016	J. ClinAnesth. 2016; 34: 350–351; [16]	No data		No data	Levodopa+ Benserazide	Pyridostigmine 60 mg × 4/d
2016	Geriatr. Gerontol. Int. 2016; 16 (4): 528–530; [17]	Increased jitter and decrement > 20%	Negative Anti-AchR	Positive Neostigimine test	Levodopa	1. Pyridostigmine 60 mg × 3/d + Prednisone 30 mg q.d.+ AZA 100 mg q.d 2. After one week ivIg 0.5 mg/kg was started—led to improvement
2016	J. Neurol. Disord. 2016, 4:4; [18]	Decrement 3 Hz	anti-AchR positive	Positive Neostigimine test	Levodopa	Pyridostigimine 120 mg —significant improvement
2016	JAmGeriatr Soc. 2016;64 (10): e120–e122; [19]	Decrement 3 Hz in trapezius	anti-AchR positive	Positive Edrophonium test	Levodopa+ Carbidopa	Pyridostigmine 60 mg q.d.+ Prednisone 30 mg q.d.—significant improvement
2017	J. Neurol. Sci. 2017; 376: 216–218; [20]	Myopathic pattern	anti-AchR positive	Not performed	Levodopa	1. Pyridostigmine led to dysphagia and dysarthria improvement, but not in the head drop; 2. ivIg—led to no benefit Steroids not tried
2018	Nervenartz. 2018:89 (4): 443–445; [21]	No decrement in all four cases	anti-AchR positive	Positive Edrophonium test in all cases		1.—Pyridostigmine 240 mg q.d + Prednisolone 20 mg q.d + AZA 2.5 mg/kg/d followed by ivIg; 2.—Pyridostigmine 330 mg q.d. + Prednisolone 20 mg q.d; 3.—Pyridostigmin 120 mg q.d.+ Prednisolone 10 mg—significant improvement; 4.—Pyridostigmine bromide 210 mg q.d. + ivIg for five days (0.4 g/kg)—significant improvement
2019	Clin. Neurol. Neurosurg. 2019; 179: 1–3; [22]	Decrement 3Hz in deltoid muscle	anti-AchR positive	Positive Ice and Intrastigimine test	Levodopa + Carbidopa	Pyridostigmine + Prednisone +AZA

Abbreviations: AZA—Azathioprine; ivIg—intravenous immunoglobulin; THP—Trihexyphenidyl; q.d.—quaque die (once a day); Ab—antibody; AchR—acetylcholine receptor; ADM—abductor digiti minimi; Anti-MusK—anti-muscle specific tyrosine kinase; MG—Myasthenia Gravis; PD—Parkinson’s disease; Hz—Herz.

## Abbreviations

Ab	antibody
AchR	acetylcholine receptor
ADM	abductor digiti minimi
Anti-MusK	anti-muscle specific tyrosine kinase
MG	Myasthenia Gravis
PD	Parkinson’s disease
REM	rapid eye movement
MMSE	Mini Mental State Examination
MoCA	Montreal Cognitive Assessment
EMG	electromyography
THP	Trihexyphenidyl
ivIg	intravenous immunoglobulin
AZA	Azathioprine
PET	positron emission tomography
GM1 ganglioside	monosialotetrahexosylganglioside
q.d.	quaque die (once a day)
b.i.d.	bis in die
t.i.d.	ter in die
